# Supplementary material for: A scoping review of regulatory T cell dynamics in convalescent COVID-19 patients – indications for their potential involvement in the development of Long COVID?
Source: Front Immunol. 2022 Dec 13;13:1070994. doi: 10.3389/fimmu.2022.1070994 (PMC9792979; doi:10.3389/fimmu.2022.1070994)
Supplement: Supplementary file 1 [file Table_1.docx]

Supplementary Material

# Search syntax

Pubmed:

*("COVID-19"[Mesh] OR "COVID-19"[tw] OR "COVID 19"[tw]) AND ("Treg*"[tw] OR "regulatory T*"[tw] OR "T regulatory cell*"[tw] OR "T-regulatory cell*"[tw] OR "foxp3*"[tw] OR "T-Lymphocytes, Regulatory"[Mesh]) NOT (Review[pt])*

Web of Science:

*TS=("COVID-19") AND TS=("Treg*" OR "regulatory T*" OR "T regulatory cell*" OR "T-regulatory cell*" OR "foxp3*")*
